# Microbiome heterogeneity in tissues of the coral, *Fimbriaphyllia* (*Euphyllia*) *ancora*

**DOI:** 10.1111/1758-2229.13310

**Published:** 2024-07-09

**Authors:** Po‐Shun Chuang, Tzu‐Haw Wang, Chih‐Ying Lu, Kshitij Tandon, Shinya Shikina, Sen‐Lin Tang

**Affiliations:** ^1^ Biodiversity Research Center, Academia Sinica Taipei Taiwan; ^2^ Institute of Oceanography National Taiwan University Taipei Taiwan; ^3^ Molecular and Biological Agricultural Sciences Program, Taiwan International Graduate Program National Chung Hsing University and Academia Sinica Taipei Taiwan; ^4^ Graduate Institute of Biotechnology National Chung Hsing University Taichung Taiwan; ^5^ School of BioSciences The University of Melbourne Melbourne Victoria Australia; ^6^ Institute of Marine Environment and Ecology National Taiwan Ocean University Keelung Taiwan; ^7^ Center of Excellence for the Oceans National Taiwan Ocean University Keelung Taiwan; ^8^ Taiwan Ocean Genome Center National Taiwan Ocean University Keelung Taiwan

## Abstract

Coral microbiomes differ in the mucus, soft tissue and skeleton of a coral colony, but whether variations exist in different tissues of a single polyp is unknown. In the stony coral, *Fimbriaphyllia ancora*, we identified 8,994 amplicon sequencing variants (ASVs) in functionally differentiated polyp tissues, i.e., tentacles, body wall, mouth and pharynx, mesenterial filaments, and gonads (testes and ovaries), with a large proportion of ASVs specific to individual tissues. However, shared ASVs comprised the majority of microbiomes from all tissues in terms of relative abundance. No tissue‐specific ASVs were found, except in testes, for which there were only two samples. At the generic level, *Endozoicomonas* was significantly less abundant in the body wall, where calicoblastic cells reside. On the other hand, several bacterial taxa presented significantly higher abundances in the mouth. Interestingly, although without statistical confirmation, gonadal tissues showed lower ASV richness and relatively high abundances of *Endozoicomonas* (in ovaries) and *Pseudomonas* (in testes). These findings provide evidence for microbiome heterogeneity between tissues within coral polyps, suggesting a promising field for future studies of functional interactions between corals and their bacterial symbionts.

## Graphical abstract

The stony coral, *Fimbriaphyllia (Euphyllia) ancora*, demonstrates microbiome heterogeneity across its functionally differentiated tissues. Several bacteria, including *Endozoicomonas* and *Pseudomonas*, exhibit differential abundances among these tissues. These findings not only broaden our understanding of coral‐bacteria symbiosis but also highlight the potential use of probiotics to enhance coral reproduction.

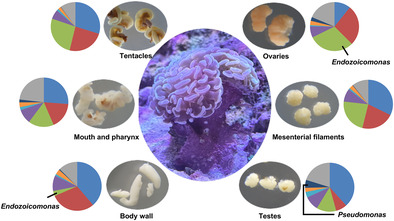

## INTRODUCTION

Corals form symbioses with a great diversity of microorganisms, including photosynthetic dinoflagellates, bacteria, archaea, viruses, and fungi, collectively termed the coral holobiont. Bacterial symbionts, sometimes simply referred as the coral microbiome, serve various critical functions in the coral holobiont, such as nutrient recycling and synthesis of vitamins and antimicrobial chemicals (Bourne et al., 2016; Nissimov et al., 2009; Pogoreutz et al., 2022). Shifts in the bacterial community in bleached or diseased corals suggest links between microbiomes and coral physiology. In addition, biochemical and genomic studies have demonstrated DMSP (dimethylsulfoniopropionate) degradation or biosynthetic capacity in several coral‐associated bacteria (Chiou et al., 2023; Doering et al., 2023; Kuek et al., 2022; Raina et al., 2009; Tandon et al., 2020). Based on metagenomic and nanoSIMS (nanoscale secondary ion mass spectrometry) data, Wada et al. (2022) also revealed polyphosphate accumulation in coral‐associated microbial aggregates (CAMAs) formed predominantly by *Endozoicomonas* bacteria. These discoveries suggest that in addition to their roles in coral physiology, coral microbiomes may also participate in the biogeochemical cycles of some elements in oligotrophic reef waters.

Coral microbiomes are generally dominated by *Proteobacteria*, *Actinobacteria*, *Bacteroidetes*, *Firmicutes,* and *Cyanobacteria*, with thousands of operational taxonomic units (OTUs) often found in a single coral species (Blackall et al., 2015; Chen et al., 2011; Hong et al., 2009; Huggett & Apprill, 2019; Lee et al., 2016; Sweet et al., 2021). Coral microbiomes are specific to host species and can vary according to numerous factors, such as the physiological conditions of coral hosts, geographic locations, and environmental fluctuations (Ainsworth et al., 2010; Glasl et al., 2019; Sunagawa et al., 2010; Ziegler et al., 2019). In addition, coral microbiomes are heterogeneous within a colony. Distinct microbial communities reside in different compartments of a coral colony, i.e., the coral surface mucus layer, soft tissue, and skeleton (Lee et al., 2016; Pollock et al., 2018; Sweet et al., 2011; Tandon et al., 2023). Moreover, factors such as light intensity, water movement, dissolved oxygen level, and even chemical components, can vary greatly between sublocations of a coral colony, e.g., the top and bottom sides of a colony. This variation may create different microhabitats for bacteria, increasing the complexity of coral microbiomes (Hernandez‐Agreda et al., 2017).

As cnidarians, corals are diploblastic. They lack true organs and consist of two tissue layers (epidermis and gastrodermis) with a layer of extracellular matrix between them (mesoglea). Despite this simple organization, corals exhibit several locally differentiated tissues that are distributed in radial symmetry around the oral‐aboral axis, such as tentacles, mouth (actinopharynx), body wall, mesenteries, and gonads (Galloway et al., 2007; Nielsen, 2012; Ruppert et al., 2004). The coral body wall is the only tissue that is directly in contact with the calcareous skeleton, whereas tentacles contain nematocysts that are used for defence and prey capture. Once a prey item is transported through the mouth into the gastrovascular cavity, mesenterial filaments, which contain abundant glandular cells that produce digestive enzymes, degrade prey and absorb nutrients. Gonads, on the other hand, are specially differentiated tissues located in mesenteries, composed of germ cells, gonadal somatic cells and several types of neurons (Galloway et al., 2007). Given that these tissues can encounter very different extracellular conditions, such as concentrations of nutrients, oxygen, and biochemical compounds, they represent fine‐scale microhabitats in individual coral polyps. However, due to the small polyp size (1–3 mm diameter) of most scleractinian corals, it is technically challenging to investigate microbiomes in specific tissues.

The anchor coral, *Fimbriaphyllia ancora* (Cnidaria, Anthozoa, Scleractinia, Euphylliidae), formerly called *Euphyllia ancora* (Luzon et al., 2017), is a common stony coral in the Indo‐Pacific Ocean, including reefs around Taiwan. Its relatively large polyp size (3–5 cm diameter) allows the microscopic isolation of specific tissues for histological and molecular studies (Chiu et al., 2019; Shikina et al., 2013, 2016; Shikina, Chiu, et al., 2015). In the past two decades, the reproductive biology of *F. ancora* has been intensively studied in Taiwan, from the possible role of sex steroids in coral spawning (Twan et al., 2003, 2006) to gametogenesis (Chiu et al., 2020; Shikina et al., 2012, 2020; Shikina, Chung, et al., 2015). In the present study, we employed *F. ancora* for a survey of bacterial communities in different coral tissues. Tissue‐specific microbiomes have been reported in some other marine invertebrates, which were attributed to differences in biological functions and/or cellular components (Dubé et al., 2019; Høj et al., 2018; Meisterhans et al., 2016). Given that *F. ancora* exhibits tissue‐specific gene and protein expression (Chiu et al., 2019; Shikina et al., 2016; Shikina, Chung, et al., 2015), we hypothesized that preferential bacterial colonization occurs in different tissues in *F. ancora*. The results of this study are expected to illuminate microbial heterogeneity in single coral polyps and provide valuable insights into functional interactions between corals and associated microbiomes.

## EXPERIMENTAL PROCEDURES

### Sample collection

**FIGURE 1:**
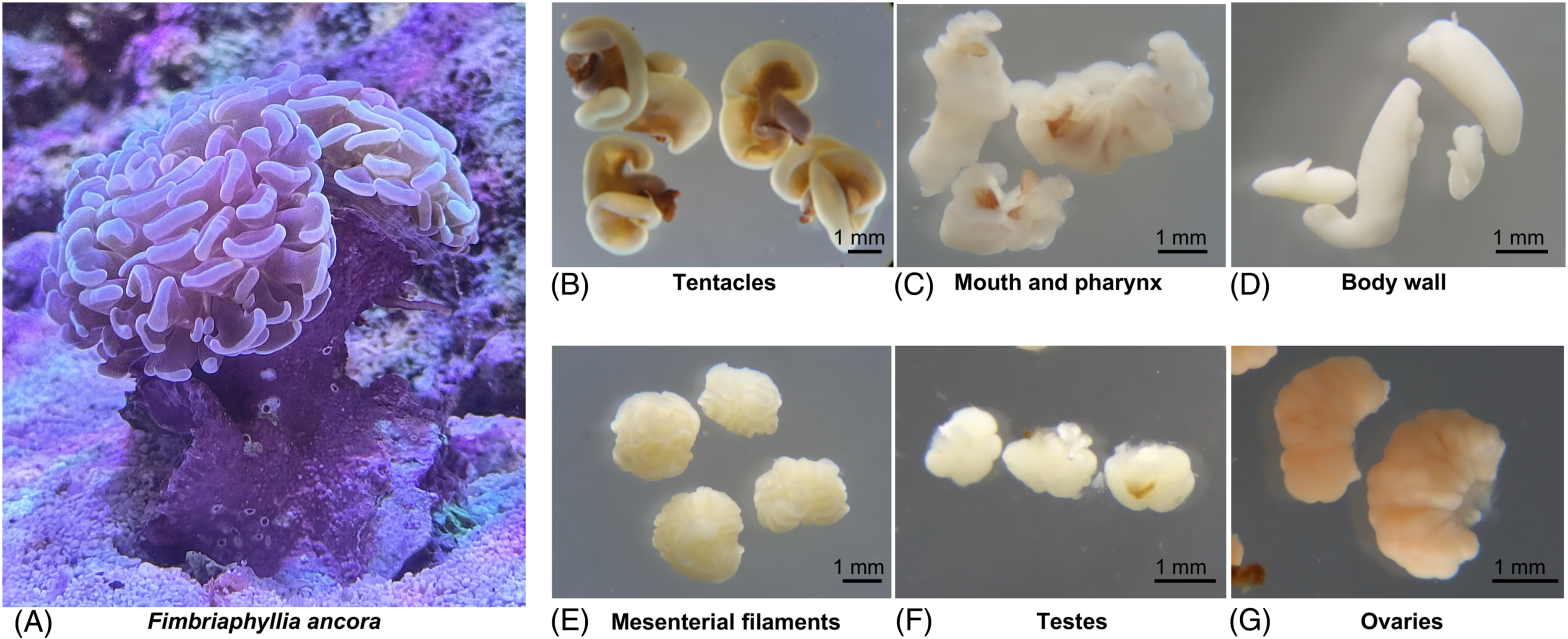
*Fimbriaphyllia ancora* and isolated polyp tissues examined in this study. (A) *Fimbriaphyllia ancora* polyp. (B) Tentacles. (C) Mouth and pharynx. (D) Body wall. (E) Mesenterial filaments. (F) Testes. (G) Ovaries.

In September 2017, we collected coral polyps from three *F. ancora* colonies at about 10 m depth at Nanwan Bay, Kenting National Park, Kenting, Taiwan (21°57′ N, 120°46′ E). Two polyps were collected on opposite tips of the longest axis of each colony to sample more of the variations among colony sublocations. Collected polyps were fixed with 20% Zinc Formal‐Fixx (Thermo Shandon, Pittsburgh, PA, USA) for 12–24 h and then rinsed twice with 0.22 μm‐filtered, sterilized seawater. Fixed coral polyps were carefully dissected using sterilized forceps to collect tentacles, mouths, body walls, mesenterial filaments, and gonads (Figure 1), following the approach of Shikina et al. (2013). Two replicates were collected for all tissue types in each polyp except the mouth, for which tissue was only sufficient for one sample. This resulted in 54 samples comprising duplicates of multiple tissue types (except for the mouth) from six polyps originating from three colonies. Tissue samples were immediately frozen in liquid nitrogen and stored at −80°C until DNA extraction.

### DNA extraction, 16S rRNA library preparation, and sequencing

Frozen tissue samples were homogenized with sterile mortars and pestles and resuspended in TE buffer (10 mM Tris–HCl, pH 7.5; 1 mM disodium‐EDTA pH 8.0) for total DNA extraction using a modified CTAB method (Hong et al., 2009; Wilson, 2001). The bacterial 16S rRNA gene was amplified using a primer pair specific for the V6‐V8 hypervariable region: 968F (5′‐AACGCGAAGAACCTTAC‐3′) and Uni1391R (5'‐ACGGGCGGTGWGTRC‐3′) because this 16S rRNA region captures higher bacterial diversity than the commonly used V4‐V5 hypervariable region (Willis et al., 2019). PCR was performed using a T100 Thermal Cycler (Bio‐Rad, UK) with the following program: an initial step at 94°C for 5 min; 30 cycles of 94°C for 30 s, 52°C for 20 s and 72°C for 45 s, with a final extension at 72°C for 10 min. Target PCR products (~450 bp) were recovered using a QIAEX II Gel Extraction Kit (Qiagen, Taiwan) following the manufacturer's instructions. DNA‐tagging PCR was then carried out following the protocol in Chen et al. (2011). Concentrations of tagged PCR products were determined with a Qubit dsDNA HS assay (Invitrogen, USA). Equal amounts of tagged PCR products for all tissue samples were pooled as 2 libraries and submitted for 2 × 250 bp pair‐end Illumina MiSeq sequencing, performed by Yourgene Bioscience (Taipei, Taiwan).

### MiSeq sequencing data processing

Pair‐end reads from Illumina MiSeq sequencing were assembled, de‐multiplexed, trimmed and quality‐filtered using MOTHUR v1.48.0 (Schloss et al., 2009) with the following criteria: (1) exact match to barcode and primer sequences, (2) assembled contig lengths between 350 and 450 bp, (3) no ambiguous bases in the assembled contig, and (4) <8 bp continuous homopolymers in the assembled contig. Given that the same barcode was attached to both primers for each sample, de‐multiplexing was determined from the complete barcode sequence on either side of a contig. A denoising step was performed to cluster sequences with ≤2 bp difference, assuming a 0.5% sequencing error in our ~400 bp amplicons (Cheng et al., 2023). Chimeric reads searched using the *chimera.vsearch* function in MOTHUR were discarded. The remaining amplicon sequencing variants (ASVs) were then filtered for singletons (ASVs with only one count across all samples) to further reduce sequencing error. Taxonomy of ASVs was classified against the SILVA v138 database (Quast et al., 2012) at a bootstrap value threshold of 0.8 and ASVs classified as non‐bacterial, i.e., eukaryote, archaean, chloroplast, mitochondrial, or unknown were removed. Considering that sequencing depth varied greatly among libraries (11,712–207,320 sequences/library; Figure S1) and the microbiome might not be homogeneous within the same tissue type in a polyp, libraries were first rarefied to 11,712 sequences (the smallest among all libraries). Libraries of duplicate tissue samples were then merged by pooling and re‐rarefying back to 11,712 sequences to average out the potential variation between duplicated tissue samples. The merged dataset was employed for taxonomic composition analysis. For beta analysis, the ASV abundance data was first log‐transformed. Distances between libraries were then measured as Bray–Curtis dissimilarity and visualized using the principal coordinates analysis (PCoA). However, to avoid artificial bias from data merging, alpha diversity (presented as Chao1 and Shannon indices) was measured on a per‐sample‐basis (rarefied dataset before merging) and averaged by tissue type for each polyp. Given that individual polyps from the same colony showed considerable variation (Figure S2), they were considered independent samples to explore environmental variation between sublocations of colonies. Raw datasets (un‐rarefied) and those after merging are available in Tables S1 and S2, respectively. ASV taxonomy is available in Table S3.

### Statistical analyses

Differences in alpha diversity between tissue types were examined using the Kruskal‐Wallis test, followed by post‐hoc, pairwise comparisons performed with Dunn's test (conducted in Python). For beta diversity, log‐transformed ASV abundance data were employed to test differences between tissue types using ANOSIM (1000 permutations; conducted in MOTHUR) and PERMANOVA (999 permutations; conducted using the *adonis2* function in the *vegan* package in R (Dixon, 2003)). Considering that the short amplicon (~400 bp) in this study does not allow precise taxonomic assignment at the species/strain level, relative abundance data were pooled at a generic level for the examination of inter‐tissue differences using the Kruskal–Wallis test and Dunn's test (data were log‐transformed). Given that sample sizes in this study were small, no *p*‐value adjustment was conducted for multiple comparisons.

## RESULTS

### Coral sexuality and bacterial community composition

**FIGURE 2:**
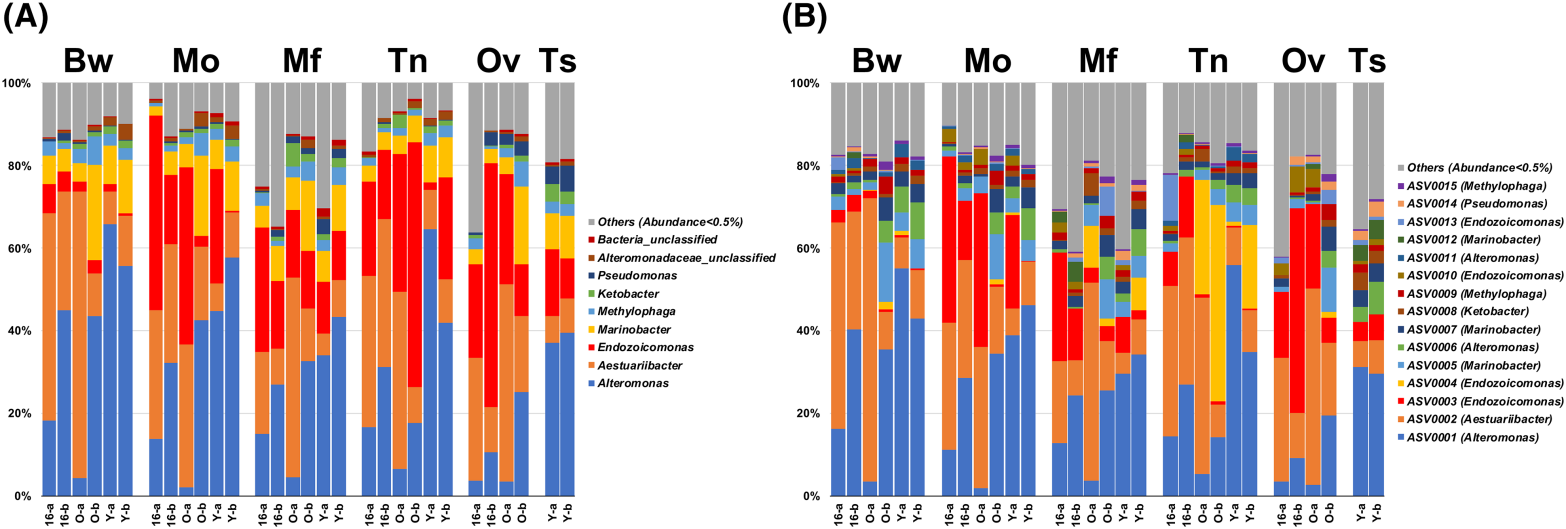
Microbiomes of *F. ancora* tissues at generic (A) and ASV levels (B). Bacterial genera and ASVs with <0.5% global relative abundances are presented as Others. The taxonomy of each ASV (at the generic level) is provided in parentheses Samples are labelled as colony ID‐polyp ID. Bw, body wall; Mf, mesenterial filaments; Mo, mouth and pharynx; Ov, ovaries; Tn, tentacles; Ts, testes.

**FIGURE 3:**
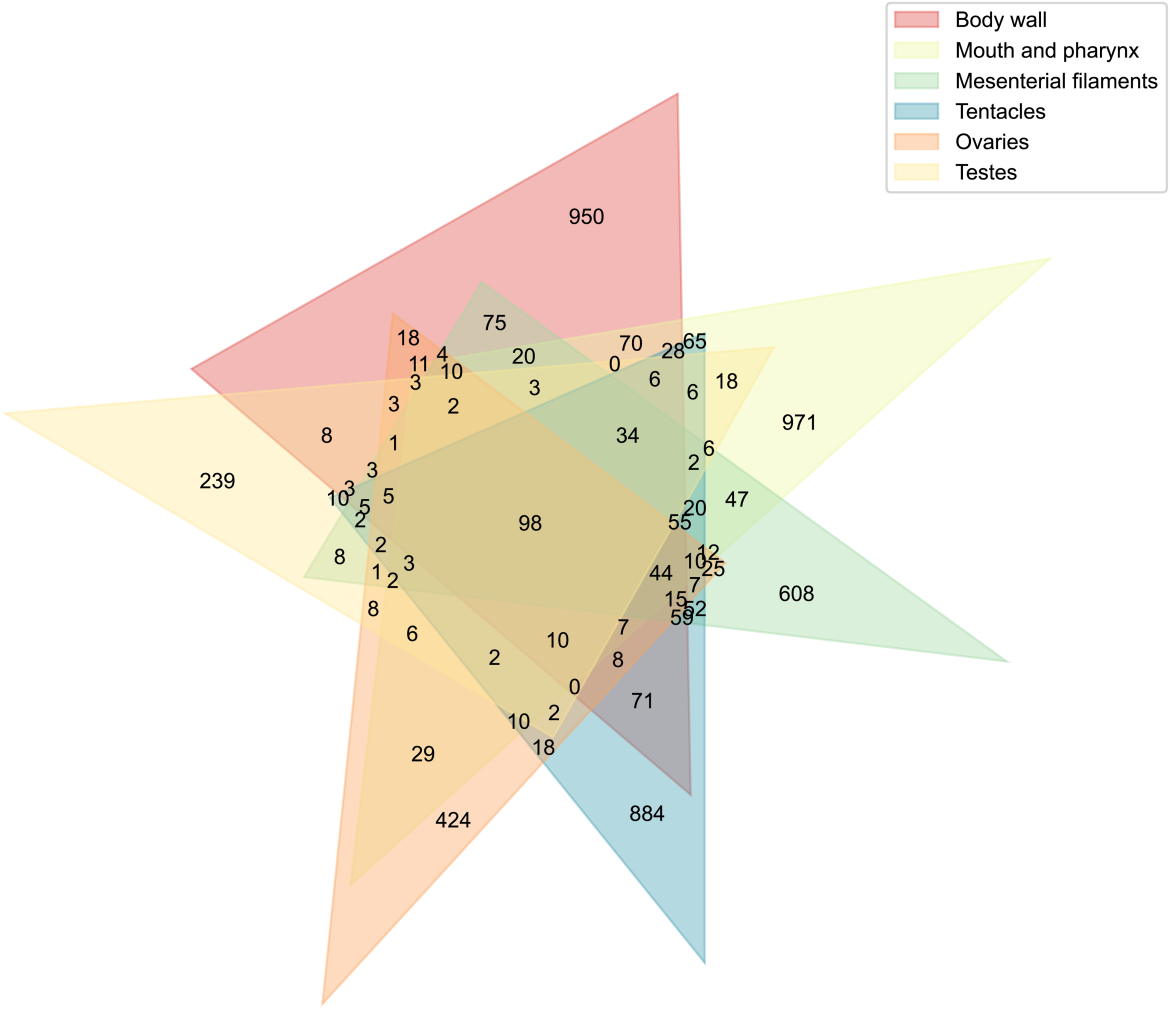
Venn diagram of bacterial ASV numbers in *F. ancora* tissues.

After microscopic dissection, the three *F. ancora* colonies were identified as two females and one male. Considering differences in developmental biology and underlying genetic mechanisms between ovaries and testes in this coral (Chiu et al., 2020; Shikina et al., 2012), the two types of gonads were treated as independent tissue types in subsequent analyses. In 54 *F. ancora* tissue samples, we obtained 5,045,526 read pairs from Illumina sequencing. MOTHUR generated 2,532,377 non‐chimeric, non‐singleton, bacterial sequences (11,712–207,320 sequences per sample), which were assigned as 8994 ASVs. Based on the SILVA v138 database, the 8994 ASVs were affiliated with 85 bacterial classes (78 known classes +8 uncultured or unclassified taxa) and 781 genera (574 known genera +207 uncultured or unclassified taxa). Data rarefaction and merging yielded 5128 ASVs from 30 merged tissue libraries, with *Alteromonas* (averaged 29.4%), *Aestuariibacter* (22.6%), *Endozoicomonas* (18.8%) and *Marinobacter* (8.6%) predominating in all libraries (Figure 2). In the merged dataset, 1629 (31.8%), 1610 (31.4%), 1240 (24.2%), 1543 (30.1%), 800 (15.6%), and 501 ASVs (9.8%) were found in body wall, mouth, mesenterial filaments, tentacles, ovaries and testes, respectively (Figure 3). A total of 98 ASVs were shared by all tissue types (1.9%; hereafter, shared ASVs), while 950 (18.5%), 971 (18.9%), 608 (11.9%), 884 (17.2%), 424 (8.3%) and 239 ASVs (4.7%) were specific to body wall, mouth, mesenterial filaments, tentacles, ovaries and testes, respectively (tissue‐specific ASVs; Figure 3). However, only 20 ASVs were conserved in all tissue types (present in over 70% samples of each tissue type) and only one tissue‐specific ASV was conserved in samples of testes. In terms of abundance, shared ASVs comprised 79.3% of the merged dataset (70.1% for the 20 conserved ASVs), whereas tissue‐specific ASVs comprised only 4.7% of the merged dataset. Relative abundances and taxonomy of the 15 most abundant ASVs are shown in Table 1.

**TABLE 1:** Mean relative abundances and taxonomy of the 15 most abundant ASVs.

ASV	Genus	Bw (%)	Mo (%)	Mf (%)	Tn (%)	Ov (%)	Ts (%)
ASV0001	*Alteromonas*	32.30	21.79	26.91	25.33	8.83	30.51
ASV0002	*Aestuariibacter*	29.24	16.93	21.09	23.62	26.45	7.11
ASV0003	*Endozoicomonas*	1.73	9.50	19.22	4.23	22.97	5.50
ASV0004	*Endozoicomonas*	0.54	3.32	0.29	16.06	0.34	0.00
ASV0005	*Marinobacter*	5.36	3.91	4.61	3.01	3.68	0.00
ASV0006	*Alteromonas*	3.86	2.60	3.01	2.22	1.17	5.71
ASV0007	*Marinobacter*	3.35	3.17	2.76	2.17	2.54	4.20
ASV0008	*Ketobacter*	1.12	1.96	1.02	1.25	0.81	3.64
ASV0009	*Methylophaga*	1.87	1.75	1.34	0.92	1.33	1.82
ASV0010	*Endozoicomonas*	0.18	0.95	1.85	0.42	3.36	1.04
ASV0011	*Alteromonas*	1.67	0.00	1.14	1.49	0.00	0.00
ASV0012	*Marinobacter*	0.21	1.21	0.02	0.28	0.32	4.25
ASV0013	*Endozoicomonas*	0.64	1.62	0.12	1.92	1.42	0.97
ASV0014	*Pseudomonas*	0.32	1.08	0.15	0.09	1.35	2.99
ASV0015	*Methylophaga*	0.84	0.76	0.68	0.48	0.60	0.51

Abbreviations: Bw, body wall; Mf, mesenterial filaments; Mo, mouth and pharynx; Ov, ovaries; Tn, tentacles; Ts, testes.

### Alpha and beta diversity

**FIGURE 4:**
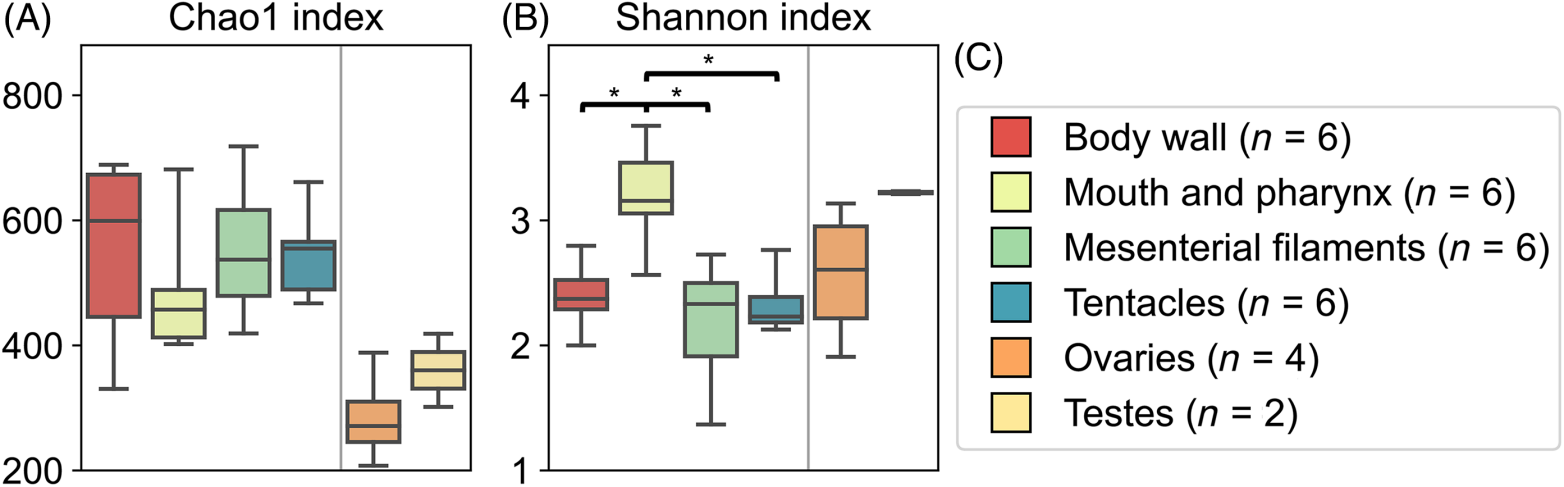
Microbiome alpha diversity of *F. ancora* tissues, calculated using the Chao1 (A) and Shannon indices (B). Pairwise comparisons were conducted for somatic tissues (separated from gonadal tissues with grey lines). Whiskers and boxes indicate the full data range and quartiles, respectively. Significant differences (Dunn's test, *p* < 0.05) are labelled with asterisks.

**FIGURE 5:**
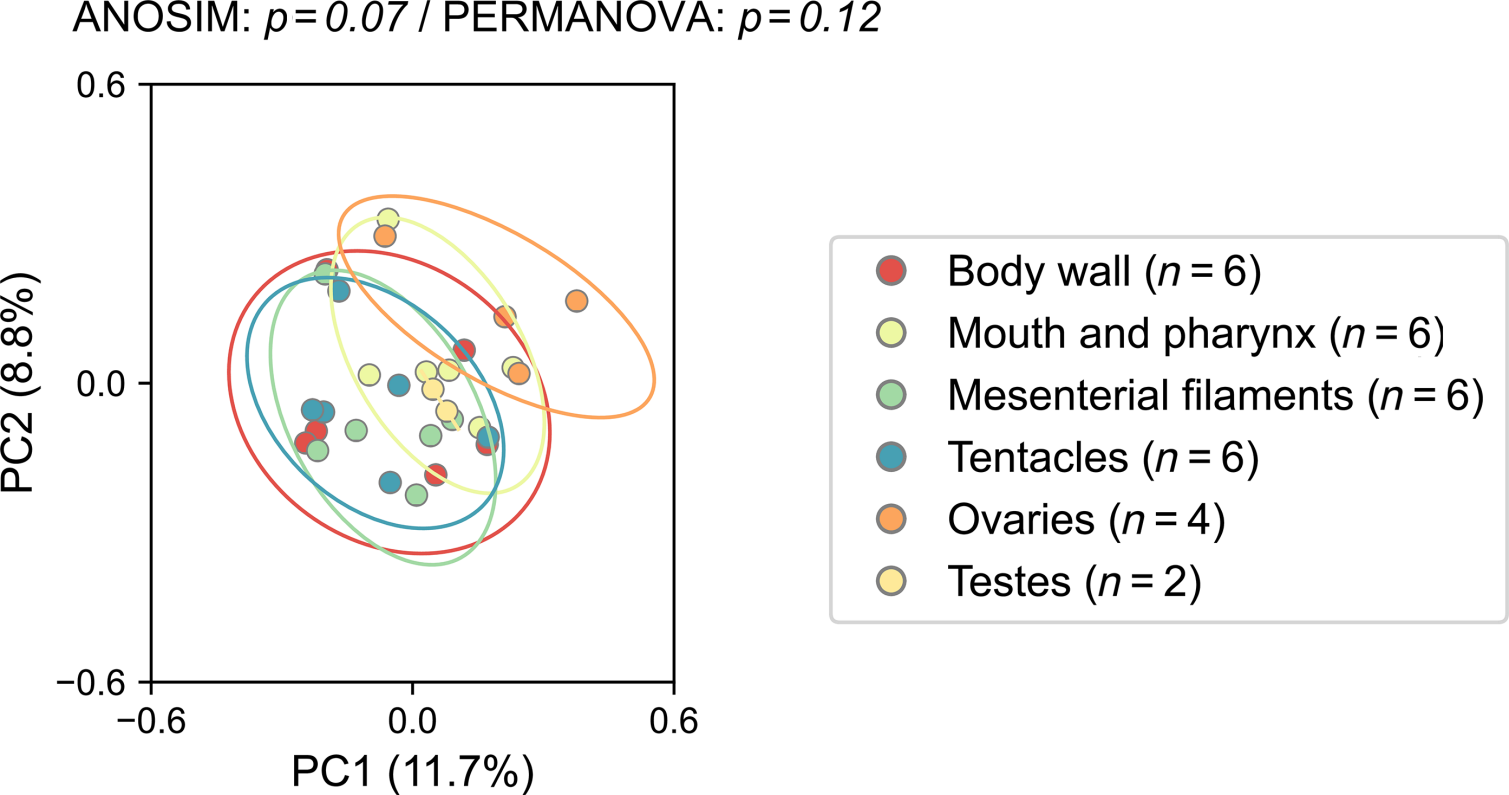
PCoA plot of *F. ancora* tissue microbiomes. Data was log‐transformed and distances between samples were calculated as Bray–Curtis dissimilarity. Circles indicate a 95% confidence interval of data distributions. No significant differences were found among somatic tissues in either ANOSIM (*p* = 0.07) or PERMANOVA (*p* = 0.12).

As ovaries and testes contained relatively fewer samples (four polyps from two colonies for ovaries and two polyps from one colony for testes), these gonadal tissues were excluded from the subsequent statistical analyses. Among the four somatic tissues, no significant difference was found for the Chao1 index. For the Shannon index, a significantly higher value was found for the mouth compared to the other three somatic tissues (Kruskal–Wallis, *p* < 0.05; Figure 4). While not included in statistical analyses, gonadal tissues showed lower values in the Chao1 index (both ovaries and testes) and higher Shannon diversity (testes) when compared to somatic tissues. For beta diversity, both ANOSIM (*p* = 0.07) and PERMANOVA (*p* = 0.12) showed no statistically significant differences among tissue types (Figure 5).

### Tissue‐associated bacteria

**FIGURE 6:**
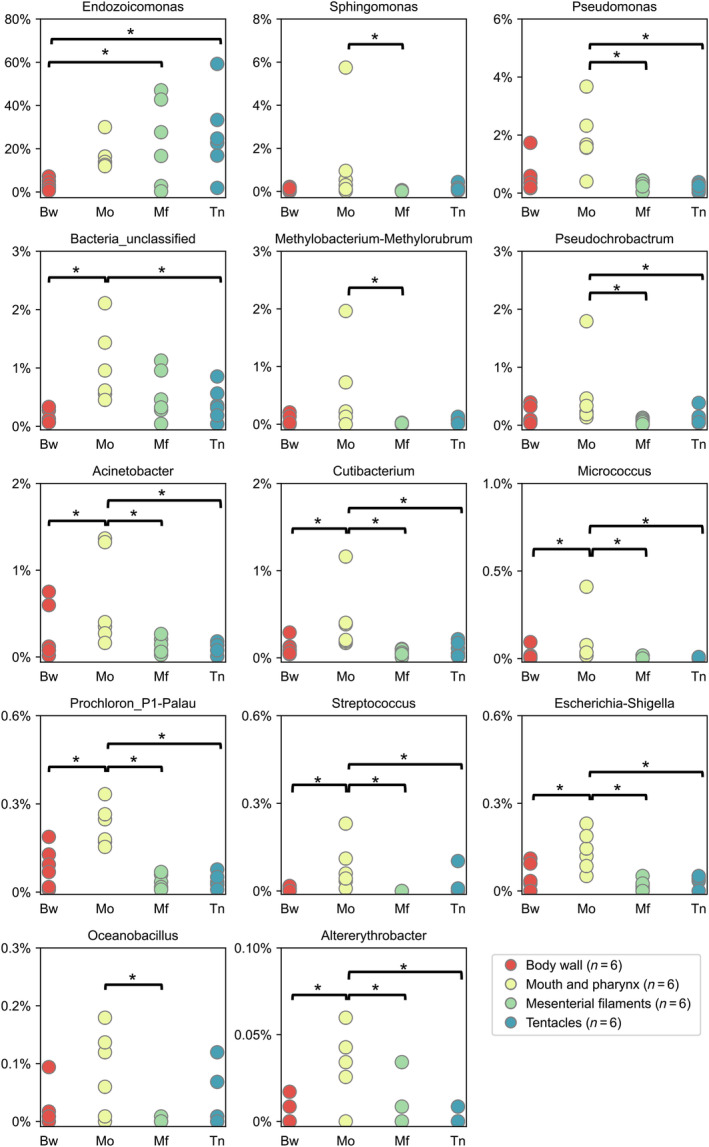
Bacterial genera exhibit significant differences among somatic tissues (Kruskal‐Wallis test, *p* < 0.05). Pairwise comparisons exhibiting significant differences (Dunn's test, *p* < 0.05) are labelled with asterisks. Bw, body wall; Mf, mesenterial filaments; Mo, mouth and pharynx; Tn, tentacles.

**FIGURE 7:**
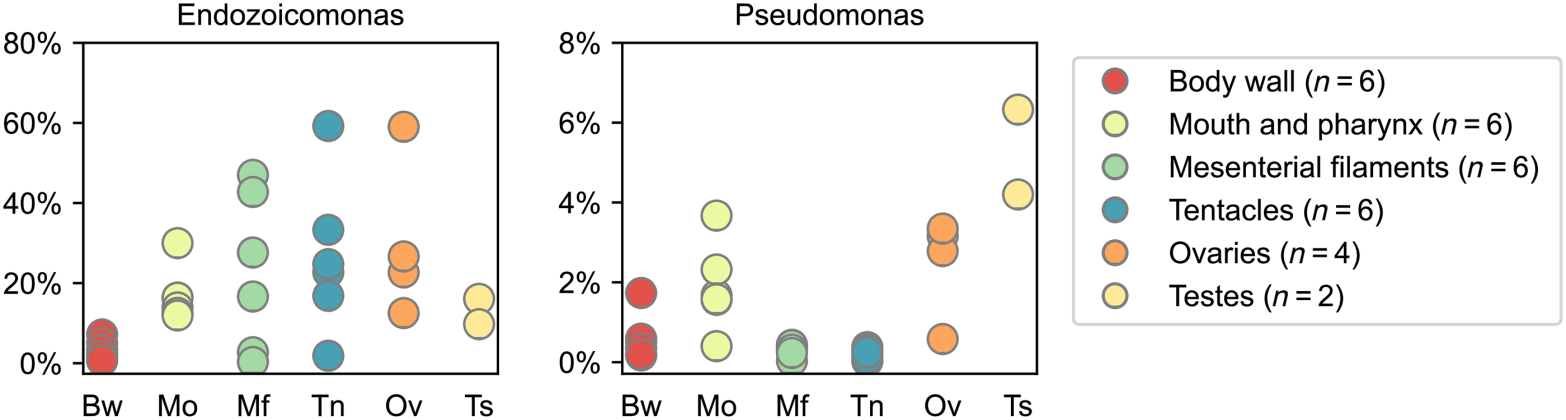
Relative abundances of *Endozoicomonas* and *Pseudomonas* in all examined tissues in this study. Bw, body wall; Mf, mesenterial filaments; Mo, mouth and pharynx; Ov, ovaries; Tn, tentacles; Ts, testes.

Among identified bacterial genera, 14 were conserved in at least one tissue type (present in >70% libraries of a given tissue type) and showed significantly different abundances between somatic tissues (Kruskal–Wallis, *p* < 0.05, Figure 6). These bacterial genera can be roughly categorized as less abundant in body walls than other somatic tissues (Group I; *Endozoicomonas*) or more abundant in the mouth than other somatic tissues (Group II; 13 genera). Although statistical tests were not applied, we also found relatively high abundances of *Endozoicomonas* and *Pseudomonas* in the two gonadal tissues (Figure 7).

## DISCUSSION

Symbiotic associations between corals and bacteria have received great attention due to their contribution to coral health and disease. Coral microbiomes may vary by environmental conditions, coral species, and even among sublocations or compartments of coral colonies (Li et al., 2014; Pollock et al., 2018; Zhang et al., 2015; Ziegler et al., 2019). However, microbiome heterogeneity between coral tissues is largely underexplored due to difficulties in isolating tissues from individual polyps. In this study, we examined bacterial communities in different polyp tissues of *F. ancora*, which has relatively large polyps. Sequencing results showed that *F. ancora* microbiomes are diverse, with about 80% of identified ASVs specific to individual tissue types and fewer than 2% being common to all tissue types (Figure 3). However, these shared ASVs comprised the majority of the bacterial community in terms of abundance (all shared ASVs: 79.3%; conserved shared ASVs: 70.1%), whereas tissue‐specific bacteria constituted only 4.7% of *F. ancora* microbiomes. Furthermore, no tissue‐specific ASV was conserved in samples of the corresponding tissue except for one ASV specific to testes (ASV0080; affiliated with *Endozoicomonas*), for which there were only two samples in our dataset. The identified tissue‐specific ASVs in this study may represent mostly opportunistic growth rather than functional symbiosis with specific coral tissues. Nevertheless, as our rarefied dataset cannot cover the full microbiomes of *F. ancora* tissues (Figure S1) and the sample preparation in this study, i.e., tissue fixing, can introduce some bias, more shared and tissue‐specific bacteria in *F. ancora* may be discovered when more data become available.

Along with the predominance of shared ASVs, beta diversity analysis showed no significant differences among tissue types examined in this study (Figure 5). Despite that, the top two components in PCoA explained only ~20% of the variation among samples in this study, suggesting the complexity of microbiomes in *F. ancora* tissues. Furthermore, several bacterial genera exhibited significantly different abundances between tissue types, including *Endozoicomonas* and several other bacterial genera (Figure 6). *Endozoicomonas* bacteria are commonly found in stony corals and their abundance has been linked to coral health and stress (Bourne et al., 2008; Chuang et al., 2024; Neave et al., 2016). Earlier studies have shown that *Endozoicomonas* bacteria form dense cellular aggregates (CAMAs) and are predominantly located in specific polyp tissues such as tentacles (in *S. pistillata*) and gastrodermal tissues (in *P. damicornis*) (Bayer et al., 2013; Neave et al., 2017; Wada et al., 2022). Our findings add further evidence for the complexity of *Endozoicomonas* spatial distribution in coral polyps. Furthermore, when compared to other tissues, body walls showed a significantly lower abundance of *Endozoicomonas*. One of the unique characteristics of body walls is the highly differentiated calicodermis, which synthesizes the calcareous exoskeletons of corals (Allemand et al., 2011; Galloway et al., 2007). Using whole transcriptomic sequencing techniques, we recently identified several highly expressed genes related to coral skeletogenesis in *F. ancora* body wall (Shikina et al., 2023). Biomineralization in corals involves the secretion of a complicated organic extracellular matrix by calicoblastic cells and delicate control of ion exchange with the extracellular calcifying medium, such as protons, Ca^2+^ and HCO_3_^−^ (Tambutté et al., 2011). Whether these molecules affect the colonization of *Endozoicomonas* is an open question for future studies.

In the mouth tissue of *F. ancora*, we found significantly higher ASV evenness and relative abundances of several bacterial genera compared to other somatic tissues. The mouth of coral polyps is where food particles enter the gastrovascular cavity and where digestive wastes and gametes are expelled to the surrounding seawater. In the mouth tissue of *F. ancora*, our recent work identified several highly expressed neuropeptides and neurotransmitters (Shikina et al., 2023). These molecules may contribute to the recruitment of specific bacteria to the mouth of *F. ancora*. Furthermore, the mouth opening constitutes a pathway for bacterial pathogens to enter and infect coral polyps, such as *Vibrio coralliilyticus* in the stony coral *P. damicornis* (Gavish et al., 2021). Preferential colonization of the mouth of *F. ancora* by specific bacteria may be linked to coral immunity. However, unlike *Endozoicomonas*, these mouth‐associated bacteria comprised only a small fraction of the microbiomes of individual tissues (Figure 6). Rare bacterial symbionts are often overlooked in microbiome studies but have been proposed to contribute significantly to coral physiology (D Ainsworth et al., 2015). The exact functional roles of these mouth‐associated bacteria in *F. ancora* warrant further investigation.

Interestingly, we observed lower Chao1 richness in *F. ancora* gonads compared to other somatic tissues (Figure 4). These findings suggest that gonadal tissues may exhibit more stringent environments than somatic tissues in corals. Steroid hormones have been reported to prime the innate immune system in eukaryotes (García‐Gómez et al., 2013; Pace & Watnick, 2021; Vom Steeg & Klein, 2017). Early studies on *F. ancora* showed increased levels of steroid hormones during spawning (Twan et al., 2003, 2006). In another stony coral, *Mussismilia harttii*, Vilela et al. (2021) showed that the estrogen, ethinylestradiol (EE2), induced significant microbial changes. Considering that our sampling was conducted at the early stages of *F. ancora* gametogenesis and only included a few gonadal samples, future studies with larger sample sizes among different stages of *F. ancora* gametogenesis should provide better insights into causation between hormonal differences and microbiome variation between gonadal and somatic polyp tissues.

Notably, *F. ancora* gonads also showed relatively high abundances of *Endozoicomonas* and *Pseudomonas* among tissues examined in this study (Figure 7). The ability to degrade steroid hormones has been proposed to mediate interactions between bacteria and symbiotic hosts (Chiang et al., 2020; Vom Steeg & Klein, 2017). Based on both genomic and experimental data, several strains of *Endozoicomonas* and *Pseudomonas* are capable of degrading testosterone (Chiang et al., 2020; Ding et al., 2016; Shintani et al., 2013; Yin et al., 1991). A gene encoding steroid delta isomerase, which converts pregnenolone to progesterone, was also found in the genome of *E. montiporae* CL‐33 (Ding et al., 2016). In fact, the 4 dominant *Endozoicomonas*‐affiliated ASVs identified in this study all matched *E. montiporae* CL‐33 when BLAST searched against the NCBI rRNA/ITS database (98.28%–100% sequence identity). These findings suggest that *Endozoicomonas* is not just preferentially associated with gonadal tissues, but may also facilitate gametogenesis in *F. ancora*. On the other hand, the dominant *Pseudomonas*‐affiliated ASV in our dataset was identical to multiple *Pseudomonas* bacteria (*P. boanensis*, *P. oleovorans* and *P. indoloxydans*). Unfortunately, none of these *Pseudomonas* taxa was originally identified in corals or seawater and no genomic data is available. Thus, no conclusions can be drawn regarding the function of *Pseudomonas* bacteria in *F. ancora*, particularly their involvement in gametogenesis. It should also be mentioned that in this study we only sequenced the V6‐V8 region of the bacterial 16S rRNA gene. Therefore, the species‐ or strain‐level taxonomy of our ASVs may be less robust and requires further confirmation.

## CONCLUSIONS

Overall, this study constituted the first survey of bacterial communities in coral polyp tissues using the stony coral, *F. ancora,* and identified microbial heterogeneity between tissues. A significantly lower abundance of *Endozoicomonas* was identified in body walls and several bacterial genera were more abundant in the mouth than in other somatic tissues. At the same time, gonadal tissues showed lower ASV richness and higher abundances of *Endozoicomonas* and *Pseudomonas* compared to other somatic tissues. These findings pave the way for future studies on functional interaction between corals and their bacterial symbionts. It should be mentioned that our samples comprise multiple polyps from each of the *F. ancora* colonies subjected in this study. Although polyps from the same colony showed great variation, their belonging to the same genet may introduce some bias. Further investigation with more samples and techniques with higher taxonomic resolution is therefore needed to provide more detailed insights.

## AUTHOR CONTRIBUTIONS

**Po‐Shun Chuang:** Validation; investigation; formal analysis; visualization; writing – original draft; writing – review and editing; conceptualization; data curation. **Tzu‐Haw Wang:** Conceptualization; data curation; formal analysis; writing – original draft; writing – review and editing; investigation; validation; visualization. **Chih‐Ying Lu:** Writing – review and editing; validation; investigation; visualization; formal analysis. **Kshitij Tandon:** Data curation; visualization; writing – review and editing; formal analysis; investigation; validation. **Shinya Shikina:** Conceptualization; methodology; funding acquisition; resources; project administration; visualization; writing – review and editing; supervision. **Sen‐Lin Tang:** Conceptualization; methodology; supervision; project administration; resources; funding acquisition; writing – review and editing.

## CONFLICT OF INTEREST STATEMENT

The authors declare that there are no conflicts of interest.

## Supporting information


**Figure S1.** Rarefaction curves of all tissue samples at the full range of sequencing depth (a) and at the first 50,000 sequences (b). The smallest sequencing depth among samples (11,712 sequencing/library) is highlighted with black dashed lines.

**Figure S2.** Pearson correlation coefficient of merged tissue samples. Analysis was based on log‐transformed ASV abundance data.

**Table S1.** Raw ASV abundance table before rarefaction.

**Table S2.** Merged ASV abundance table. Sequence depth is rarefied to 11,712 sequences.

**Table S3.** Taxonomy of all ASVs generated in this study.

## Data Availability

All sequencing data generated in this study are available on the National Center for Biotechnology Information (NCBI) Sequence Read Archive (SRA) database under BioProject PRJNA720602.
