# Supplementary figures and images for: Microbiome heterogeneity in tissues of the coral, Fimbriaphyllia (Euphyllia) ancora
Source: Environ Microbiol Rep. 2024 Jul 9;16(4):e13310. doi: 10.1111/1758-2229.13310 (PMC11233273; doi:10.1111/1758-2229.13310)

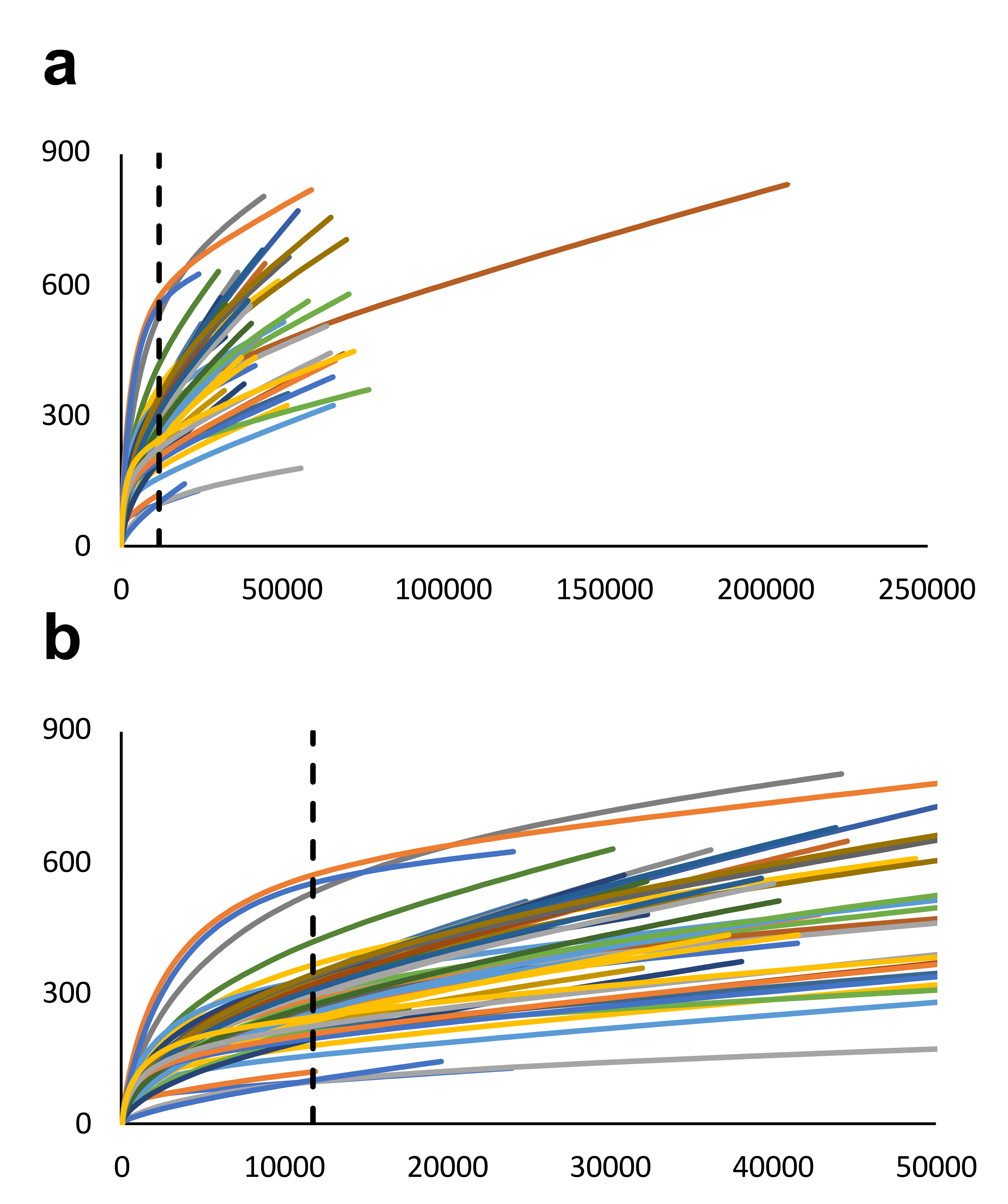

Supplement: Supplementary file 1 — Figure S1. Rarefaction curves of all tissue samples at the full range of sequencing depth (a) and at the first 50,000 sequences (b). The smallest sequencing depth among samples (11,712 sequencing/library) is highlighted with black dashed lines. [file EMI4-16-e13310-s004.tiff]

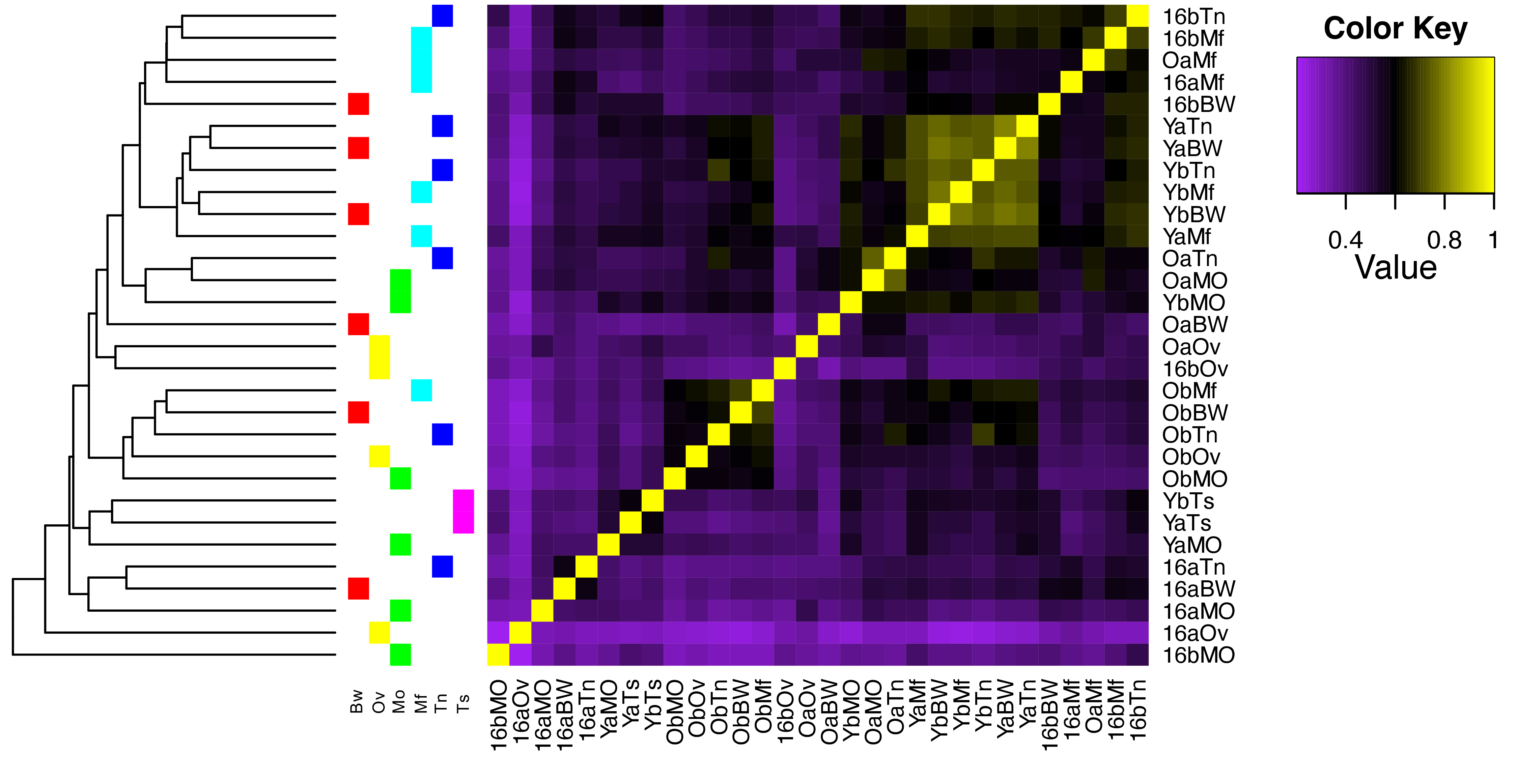

Supplement: Supplementary file 2 — Figure S2. Pearson correlation coefficient of merged tissue samples. Analysis was based on log‐transformed ASV abundance data. [file EMI4-16-e13310-s001.tiff]
